# Transformative effect of a Humanitarian Program for individuals affected by rare diseases: building support systems and creating local expertise

**DOI:** 10.1186/s13023-022-02192-1

**Published:** 2022-04-04

**Authors:** I. C. Verma, A. El-Beshlawy, A. Tylki-Szymańska, A. Martins, Y.-L. Duan, T. Collin-Histed, M. Schoneveld van der Linde, R. Mansour, V. C. Dũng, Pramod K. Mistry

**Affiliations:** 1 Institute of Genetics and Genomics, Sir Ganga Ram Hospital, Rajinder Nagar, New Delhi, 110060 India; 2 Department of Pediatrics, Faculty of Medicine, Cairo University, Cairo, Egypt; 3 Department of Pediatrics, Nutrition and Metabolic Diseases, The Children’s Memorial Health Institute, Warsaw, Poland; 4 Fabry Registry Brazil, Latin America Fabry, Gaucher, MPS I and Pompe Registries, Universidade Federal de São Paulo, São Paulo, Brazil; 5 Medical Oncology Department, Pediatric Oncology Center, Beijing Children’s Hospital, Capital Medical University, National Center for Children’s Health, Beijing, China; 6 Beijing Key Laboratory of Pediatric Hematology Oncology, Key Laboratory of Major Diseases in Children, Ministry of Education, Beijing, China; 7 International Gaucher Alliance, Dursley, UK; 8 Independent patient author, Varsseveld, The Netherlands; 9 Project HOPE (Health Opportunities for People Everywhere), Cairo, Egypt; 10 Department of Medical Genetics, Metabolism and Endocrinology, Vietnam National Children’s Hospital, Hanoi, Vietnam; 11 Department of Internal Medicine, Yale Liver Center, Yale University School of Medicine, 333 Cedar Street, PO Box 208019, New Haven, CT 06520 USA

**Keywords:** Humanitarian Program, Charitable program, Rare diseases, Lysosomal storage disorders, Enzyme replacement therapy

## Abstract

Rare diseases affect > 400 million people globally with a disproportionate burden falling on children, resulting in high morbidity and mortality rates. Affected individuals in some under-resourced countries have limited access to expert care or treatments; moreover, they suffer long diagnostic journeys during which debilitating and life-threatening complications occur. Lysosomal storage disorders (LSD) are prototype rare diseases due, in the main, to inherited deficiencies of lysosomal enzymes/transporters that affect up to 1 in 5000 newborns. Recognizing the need to provide treatment access to people with LSDs everywhere, a collaborative partnership was pioneered and set up 30 years ago. Partnering with local authorities, non-government organizations across six continents, local as well as international experts, a robust, sustainable Humanitarian Program emerged that now represents the most enduring charitable access program for LSD treatment. Here we present the history, process, lasting beneficial effect of the program to develop healthcare systems and infrastructures, and the lessons learned from addressing major unmet needs for LSDs.

## Background

Rare diseases affect > 400 million people globally, impacting more individuals than cancer and acquired immune deficiency syndrome (AIDS) combined [1]. These individuals have little or no access to healthcare and undergo protracted diagnostic journeys, enduring chronic disability and life-threatening complications [2], while being vulnerable to stigma and local cultural prejudices. In some regions, a disproportionate burden of disease falls on the children of resource-poor populations, resulting in high morbidity and premature mortality [1].

Lysosomal storage disorders (LSDs) are prototype rare diseases that are, for the most part, due to deficiencies in one of the lysosomal enzymes/transporters [3, 4], with a combined incidence of up to 1 in 5000 newborns [5]. The accumulation of toxic substrates in the lysosomes results in cellular dysfunction, multisystem organ damage, and heterogeneous disease manifestations in children and adults (Tab l e 1) [3, 4].

**Table 1:** Clinical manifestations of selected lysosomal storage disorders [3, 4]

Disease	Subtypes	Gene	Treatment	Enzyme	Substrate	Clinical manifestations
Gaucher disease	Three clinical phenotypes: Type I, which does not have neurologic involvement Types II and III, the so-called neuronopathic forms, which both feature neurologic impairment	*GBA*	Imiglucerase—(Gaucher disease type I and type III) Alglucerase—(Gaucher disease type I) Eliglustat tartrate—(Gaucher disease type I)	Glucocerebrosidase	Glucosylceramide	Hepatosplenomegaly, thrombocytopenia, anemia, bone pain, and poor growth in children
Fabry disease	Age of onset is highly variable and can range from early childhood to the fifth decade or later Classic disease Late onset disease – milder form	*GLA*	Agalsidase beta	Alpha-galactosidase A/Alpha-galactosidase	Globotriaosylceramide (GL3)	Neuropathic pain, gastrointestinal symptoms, angiokeratomas (clusters of purplish, non-blanching punctate lesions) and hypohidrosis, and deteriorating renal function
Pompe disease	Infantile onset Pompe disease	*G AA *	Alglucosidas e alfa	Alglucosidase alfa	Glycogen	Hypertrophic cardiomyopathy, respiratory insufficiency, respiratory failure, muscle weakness, feeding/swallowing difficulties, hypotonia, and developmental delay
	Late onset Pompe disease					Limb-girdle weakness, respiratory insufficiency, feeding/swallowing difficulties, gastrointestinal symptoms, ptosis
MPS I	Seven distinct forms (I, II, III, IV, VI, VII, IX) and numerous subtypes (eg, IIIA, IIIB, IIIC, IIID)	MPS I: *IDUA*	Laronidase	Alpha-L-iduronidase	Mucopolysaccharides (eg, dermatan sulfate, heparan sulfate, keratan sulfate)	Developmental delay, organomegaly, and dysostosis multiplex
		MPS II: *IDS*	Idursulfase	Iduronate sulfatase		
		MPS VI: *ARSB*		Arylsulfatase B		

ARSB, arylsulfatase B precursor; GAA, acid alpha-glucosidase; GBA, glucocerebrosidase; GLA, galactosidase alpha; IDUA, alpha-L-Iduronidase; MPS, mucopolysaccharidosis

Although recombinant enzyme replacement therapies (ERT) are available for increasing numbers of these disorders, it is challenging for affected individuals in under-served populations to access treatment due to geographical location, local healthcare infrastructure, and prohibitive costs [6].

**Fig. 1:**
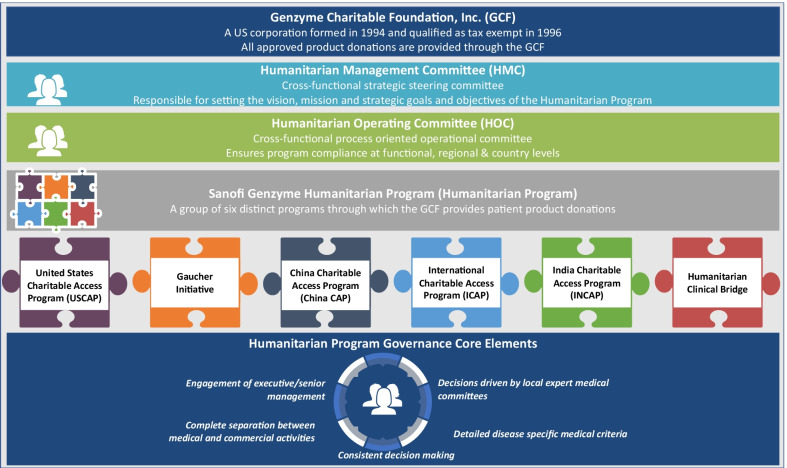
Governance structure of the Humanitarian Program

**Fig. 2:**
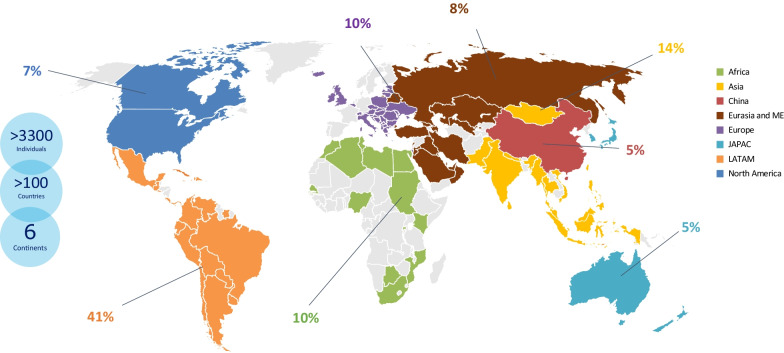
Percentage of individuals enrolled into the Humanitarian Program by region historically. JAPAC, Japan and Asia–Pacific; LATAM, Latin America; ME, Middle East

Thirty years ago, a collaborative Humanitarian Program was pioneered by Genzyme (now Sanofi Genzyme) (Fig. 1) based on the premise that individuals with LSDs should have access to treatment regardless of circumstance. This mission was fulfilled through the pioneering of a robust, sustainable Humanitarian Program in partnership with local authorities and non-government organizations (NGO) across six continents [7]. To date, the program has provided treatment for > 3300 individuals in more than 100 countries, some of whom have received treatment for > 20 years **(**Fig. 2) for the LSDs Gaucher disease (GD), Fabry disease (FD), Pompe disease (PD), and mucopolysaccharidosis (MPS) type I and II (Table 2).

**Table 2:** Individuals enrolled in the Humanitarian Program by disease

Disease	Year disease introduced to program	Historical data		Current data^b^			
		Number of individuals since inception	Number of countries supported since inception	Number of individuals receiving humanitarian treatment annually	Number of countries supported today	Average number of new cases approved each year; over past 5 years	Average time current individuals have received humanitarian treatment (years)
Gaucher disease	1991	1248	75	652	52	48.2	9.8
Fabry disease	2003	972	50	150	21	59.2	3.4
Pompe disease	2006	766	70	142	37	45.8	4.8
MPS I	2003	390	55	55	20	9.8	6.6
MPS II^a^	2014	35	7	24	6	5	3.0
Total		3411	103	1023	69	168	5.5

Data as of May 2021

MPS, Mucopolysaccharidosis

^a^Humanitarian access in MPS II is limited to Sanofi Genzyme territory only

^b^Data for individuals entering the Humanitarian Program are dynamic; with a rolling submission process, new individuals are approved throughout the year. Individuals also depart the program owing to a number of reasons, including death, individual or physician decision to stop treatment, and commercial transition

The Humanitarian Program is the longest-running charitable access program (CAP) for treatment of LSDs providing sustainable global access to therapies for affected people, regardless of local healthcare system infrastructures [8]. To address the global unmet need in devastating LSDs, a new model was required, distinct from existing programs, such as those for neglected tropical diseases [8, 9]. This new model also helped develop local clinical expertise and infrastructure for diagnosis, disease monitoring, and logistics.

The program was initiated in the United States (US) in 1991 through the launch of the CAP within 3 months of alglucerase (Ceredase®) approval, the first ERT for GD type 1 [10, 11]. Individuals were accepted for compassionate treatment on a case-by-case basis, and it rapidly became apparent that a regionally focused healthcare infrastructure within countries was necessary for these rare diseases due to distinct presentations, novel phenotypes, and genotypes. Multiple regional initiatives were rolled out, culminating in the first non-US collaboration with an NGO (the Gaucher Initiative/Project HOPE [Health Opportunities for People Everywhere]). Herein, we highlight the history, process, the lasting impact of this program, and its positive effect on healthcare systems and improved infrastructure to help identify, diagnose, and manage individuals with LSDs. Specific examples of the successful program implementation are illustrated by reference to GD, but parallel initiatives for other LSDs have been established.

To assess current perceptions of the program’s impact and challenges, information was collected through a series of qualitative web-based interviews with key individuals instrumental in the program roll-out and evolution. An online survey was also conducted to evaluate operational aspects (eg, length of participation, number of individuals treated, and disruptions to treatment), experience of the program, and suggestions for improvement. The survey was open to 320 physicians with experience in treating LSDs across 70 countries. The privacy and personal data of respondents were protected in accordance with the applicable laws and terms of Global Sanofi Privacy Policy. The insights gathered and reported herein represent the collective experience of > 50,000 patient-years of treating LSDs, spanning up to 40 years, and decades-long leadership in rare-disease patient advocacy.

## Results

### Major learnings

Successful program implementation requires an infrastructure supporting treatment access, coupled with concurrent development of healthcare ecosystems. Central to this are education, development of diagnostic technologies, disease monitoring, and building of expertise in local centers to eventually develop into destination centers where individuals with LSDs can receive diagnosis, therapy and monitoring of outcomes. Success was underpinned by striving to meet the needs of all stakeholders involving seamless partnership with members of the local treating communities, patient advocacy groups and organizations, and the healthcare system.

Healthcare delivery for LSDs begins with a compassionate case-by-case response at the country level reaching far beyond simply shipping the drug. At its earliest beginnings Sanofi Genzyme recognized the essential need to build skill sets and infrastructure to support complex care for these people. Individual physician training rapidly evolved into medical advisory boards of enterprising local physicians, international expert physicians, and humanitarian initiative staff. These forums served as platforms for training and mentorship by international leaders, such that over time local physicians became thought leaders advancing the science and generating new knowledge in these rare diseases. The medical advisory boards collaboratively developed treatment guidelines, monitoring, and stratification of individuals based on disease pattern, severity, and genotypes. Over time, local expert physicians took increasing leadership in individuals’ selection, considering nuances of disease phenotypes, and treatment plans. Often, the phenotypes encountered had no prior counterpart in world literature, which is primarily European-centric. Technological developments included logistics of cold-chain transport of medication, advanced assessments to monitor the individuals, and transfer of knowledge and capabilities from a country with limited resources to a central laboratory in another country. For example, the development of dry-blood-spot technology for biochemical molecular analysis, and biomarkers studies in LSDs, advanced monitoring of individuals in remote locations without the need to ship whole blood samples to specialist laboratories. Partnership with rare-disease patient advocates and advocacy groups, renowned for their disease knowledge, was invaluable in ensuring free access to additional medical devices. Creation of such comprehensive healthcare systems supported by skilled providers is critical for improving the health of people with LSDs, and providing an established functional framework to elevate advocacy with local authorities to address the huge unmet needs of this underserved population. Achievement of these goals required overcoming disparate cultural, social, trade policy and political barriers.

### Infrastructure and standard operating procedures

**Fig. 3:**
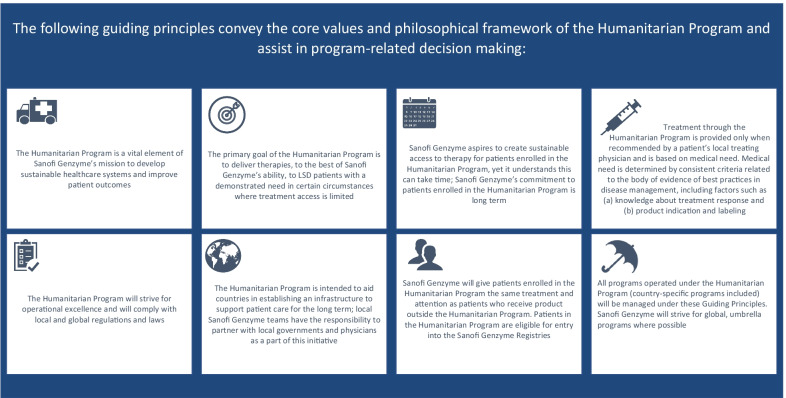
Guiding principles of the Humanitarian Program

From its inception, the governance structure has ensured the program remains compliant with ethical standards, institutional policies, and external governmental requirements, and continues to meet the strategic goals and the foundational mission of the Humanitarian Program (Fig. 3). It operates under the guidance of independent medical review boards, whose composition is region-dependent; for instance, the expert medical committee for the Gaucher Initiative comprises leading experts in GD, staff from Project HOPE and Sanofi Genzyme, and a medical ethicist [8].

Prioritization of individuals or countries is driven by the individuals’ need and is agnostic to the likelihood of government reimbursement or competitive market forces. Candidacy for treatment is primarily based on medical need in the following circumstances: when reimbursement is unavailable, until reimbursement is available, when reimbursement is available but the individual does not qualify, and when other local access or assistance programs are unavailable.

The governance structure was tailored to individual country requirements to best serve that population (Fig. 1). For example, in Egypt, as most people with GD present with massive visceral and hematologic disease in childhood, the medical advisory board had a preponderance of pediatricians and pediatric hematologists.

### Physicians’ perspectives

**Fig. 4:**
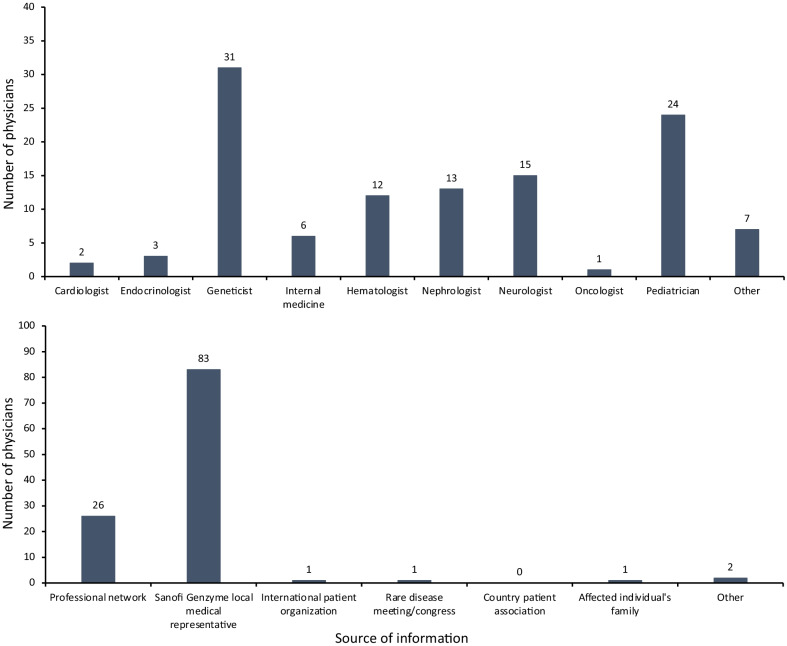
**A** Specialty breakdown of physicians participating in the survey. **B** How physicians learned about the Humanitarian Program

To evaluate the program and solicit opinions on future enhancements, 320 physicians with experience of treating LSDs were invited to take part in an online survey. Overall, 114 respondents from 42 countries completed the survey, of which 63% of physicians had individuals currently enrolled in the program. Respondents represented a broad range of specialties, including geneticists (27%), pediatricians (21%), and neurologists (13%; Fig. 4A).

Over 92% of physicians became aware of the Humanitarian Program through local or regional medical Sanofi Genzyme contacts (Fig. 4B). Two-thirds of physicians had up to five individuals that had participated in the program, with some physicians having over 20. A third (34%) of physicians reported that it took < 6 months following confirmed diagnosis for individuals to start receiving treatment, with half (54%) of all physicians reporting receiving treatments within 1 year; treatment disruptions due to COVID-19 (a lack of safe areas for infusion, country lockdowns, and travel restrictions) were noted by 35% of physicians.

Most physicians (94%) reported that individuals under their care received the scheduled treatment regularly. The remaining 6% reported challenges to treatment access, including: government regulations, challenges with storage logistics, individuals unable to comply with all the monitoring guidelines, limited financial resources for logistics of getting infusion treatment/challenging environment, delays in receiving medication, and challenges in contacting individuals.

Most physicians (92%) reported that they did not experience any problems when completing the required application forms or providing medical updates every 6 months, however, some suggestions for improvements to the program included digitizing the enrollment process, reducing the ongoing paperwork, and improving disease awareness and support.

### Examples of successful implementation

**Fig. 5:**
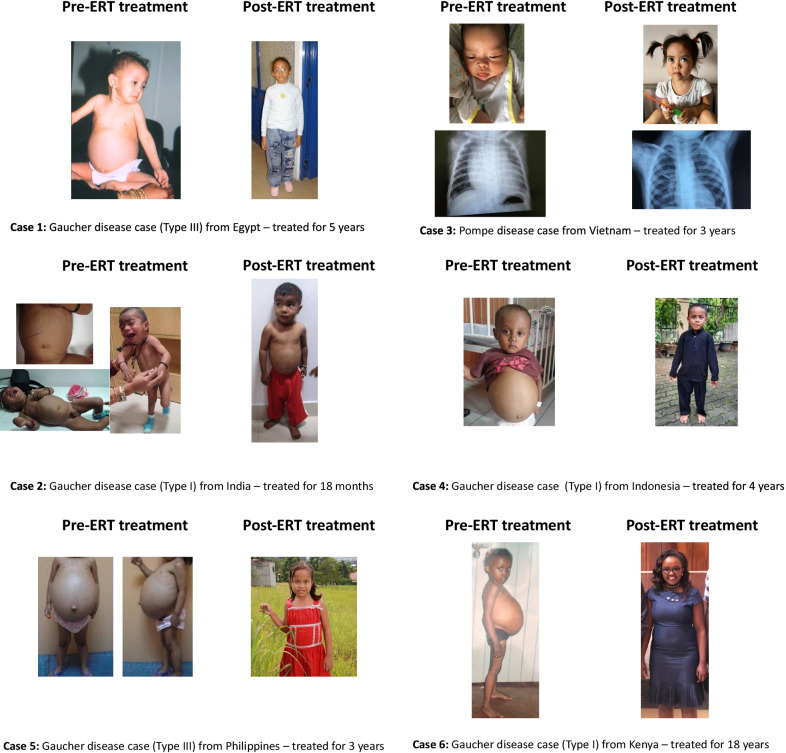
Images of individuals with rare disorders treated on the Humanitarian Program

Country-specific examples of the successful implementation to build healthcare infrastructure serving LSDs in underserved populations illustrate how, the entire clinical spectrum of GD, for example, has been redefined to serve the individual’s unique needs. Figure 5 depicts examples of individuals with LSDs who have benefited from the program. Cases 1, 2, and 4–6 show the common phenotypes of severe hepatosplenomegaly and failure to thrive pre-ERT due to GD, and resolution post-ERT. Case 3 shows infantile onset of PD with reversal of cardiomyopathy post-ERT. Of note, in some cases the benefit has extended further to the individuals’ siblings, enabling their early diagnoses and treatment.

### Egypt

The numbers of individuals with GD needing medical care became immediately evident upon inception of the program in 1999, when the number of individuals grew from 12 to 37. Project HOPE was a key partner, raising awareness of GD and assessing the gaps in treatment access. It was considered the most effective partner to manage the Gaucher Initiative, having operated in Egypt since 1975, and collaborating with the Ministry of Health and University Hospitals across the country for other healthcare initiatives. This collaboration with Project HOPE was critical in implementing many aspects of the program, including annual training for Egyptian doctors via international and national experts, and patient and family awareness activities. This initiative robustly models the key pillars of building healthcare systems, starting with affected individuals and their families, mentorship, and development of thought leaders within medical committees to achieve high-level expertise on how best to evaluate candidacy for treatment, assessing outcomes, and contributing gained knowledge to benefit the global community. To date, the program has supported > 250 individuals in Egypt with GD treated at centers of excellence established across the county, obviating the need to travel long distances for diagnosis, evaluation, and treatment.

### India

Initiated 20 years ago, the program in India has supported > 200 individuals across four LSDs. The program has resulted in improved awareness, diagnosis, and management of individuals with LSDs through the development of several centers of excellence and diagnostic capabilities, along with the implementation of medical-education initiatives for individuals with GD, FD, and PD [12–15]. Local physicians who had experience in inborn errors of metabolism expedited the rapid configuration and operation of the India Medical Advisory Board, which comprises of these experts and a group of international practitioners, chosen for their specialist knowledge of LSDs, culturally sensitive ethical considerations, and understanding of the local healthcare system. Medical educational activities and workshops played an important role in upskilling physicians caring for individuals with LSDs. There is now a cadre of expert physicians in India who are contributing new knowledge to the field.

### China

The China CAP has grown to support > 180 individuals with GD since its initiation in 1999. Since 2009, centers have spread across China, providing accurate diagnostic support and treatment for LSDs, and obviating the need to travel long distances. A growing number of provinces contribute toward costs through government medical insurance. The program’s flexibility allows for partial humanitarian support for > 50 individuals. The expert medical committee in China has developed treatment guidelines and provided country-wide clinical expertise.

### Central and Eastern Europe

Since the late 1990s individuals severely affected with GD unable to receive treatment in Central and Eastern Europe have been treated via the Humanitarian Program. Individual European countries such as Albania, Belarus, Bosnia, North Macedonia and Serbia, rapidly adopted management of individuals with LSDs, propelled by powerful advocacy groups, which has transformed care. Early access to treatment in these countries has advanced physicians’ knowledge, shortening diagnostic journeys, and improving treatment outcomes.

### Global impact

**Fig. 6:**
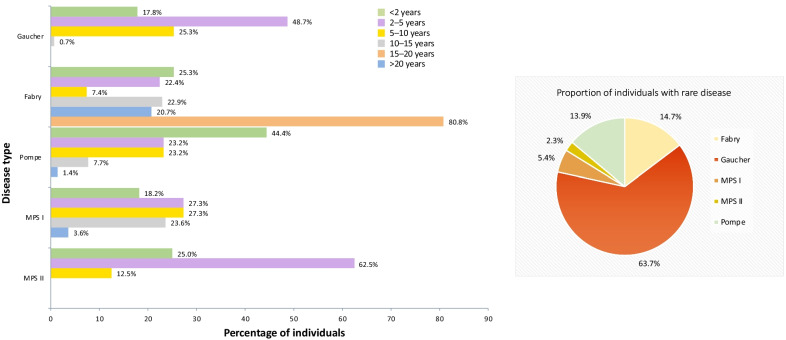
Percentage of individuals currently receiving enzyme replacement therapy in the program by time on treatment. Data as of May 2021. *Note*: humanitarian access to MPS II is limited to Sanofi Genzyme territory only. MPS, mucopolysaccharidosis

**Fig. 7:**
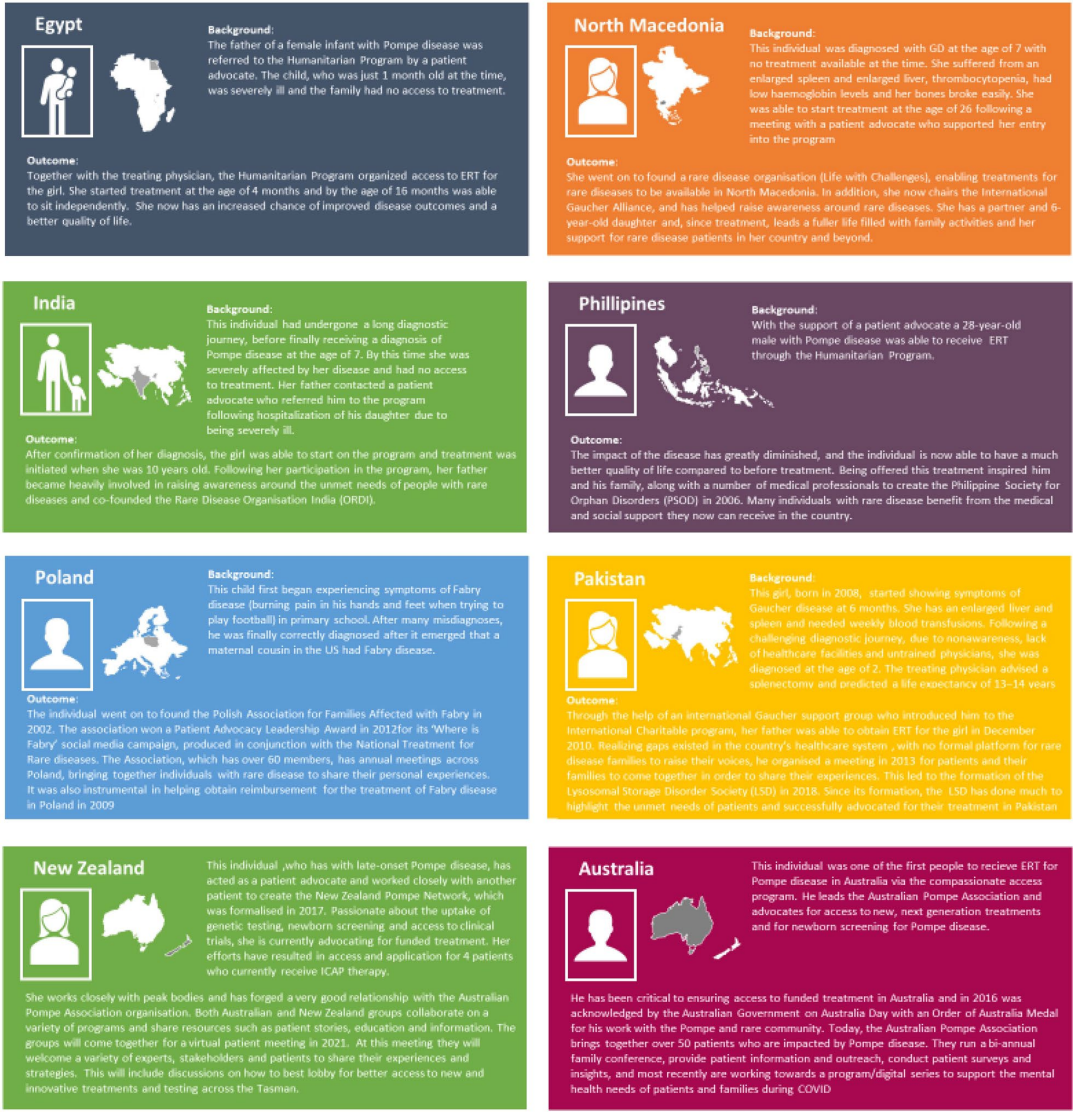
Examples of individuals with rare diseases enrolled into Humanitarian Program realizing their potential

The impact on the lives of individuals with LSDs has been transformative. Thus far, the Humanitarian Program has provided treatment to > 3300 individuals across more than 100 countries, some for over 20 years, with the average treatment duration ranging from 3 years (for MPS type II) to 9.8 years (for GD; Fig. 6). There are currently > 1000 individuals active in the program as of May 2021, with 156 individuals initiating treatment in 2020 alone. The program has also successfully allowed individuals to become disease advocates helping to increase awareness. Examples of the cascade effect of the program are demonstrated by the formations of the Organization for Rare Diseases India (ORDI), by a father of an individual with PD treated in the program in India, and the Philippine Society for Orphan Disorders (PSOD), by another individual with PD and his family in the Philippines (Fig. 7).

Experienced members of advisory boards have also played a pivotal role, with overseas experts mentoring local physicians in all aspects of LSDs. This mentorship has been central to the program, with local physicians emerging as experts contributing new knowledge to benefit the rest of the world [14, 15]; local physician communities have become highly knowledgeable in rare diseases, shortening diagnostic odysseys, and allowing for maximum clinical impact of ERT.

### Resilience and guiding principles for the Humanitarian Program in times of crisis

Operating a program of this magnitude and global reach is challenging. The lessons learned and the measures implemented to overcome obstacles at the local and global level are key to ensuring the resilience of the program.

A hallmark of the program is providing access to treatment for affected individuals who meet the medical criteria, regardless of geography. This includes sanctioned markets such as Cuba, Iran, and Sudan, where treatment access has been provided through the program for 20 years. Other examples include supporting individuals with rare diseases in Palestine, and those displaced due to war or conflict in their countries of origin, for eg, Syrian and Iraqi refugees living in Turkey. In some of these geographies, the program enlists the help of NGO partners to ensure long-term therapy and overcome barriers to treatment access. The ethical underpinnings of the program were tested during a global supply shortage of agalsidase beta in 2009 and imiglucerase in 2010 that required dose-conservation guidelines to be implemented. However, doses were rationed equitably among the most critically affected individuals and Humanitarian Program recipients to ensure that the reduced product supply was distributed based solely on medical need [16]. One of the current challenges faced by the program is the COVID-19 pandemic, which has resulted in altered relationships between international and local humanitarian organizations, deepening inequalities in access to service and the need to find creative solutions for providing access to treatment for individuals with rare disease. Nonetheless, the resiliency of the program is evident in continued treatment of affected people without interruption. Other challenges, with potential solutions, are outlined in Table 3 .

**Table 3:** Challenges encountered during the implementation and ongoing management of the Humanitarian Program

	Challenges	Solutions
Infrastructure	Lack of infrastructure for diagnosis, treatment, and management of individuals with LSDs Availability of specialists in treatment of LSDs and treatment centers	Involve all stakeholders, including external partners (individuals with rare diseases, physicians, government, patient association groups, and NGOs) and internal team members at global, regional, and local level Facilitate diagnostic services by utilizing global and regional networks in countries where local testing is not available Engage with a respected NGO with a strong local presence to help facilitate development of in-country capacities Create expert medical committees to increase local knowledge and understanding of disease awareness, diagnosis, treatment benefit, and follow-up
Medical expertise	Requirement for ongoing training and education for healthcare providers to raise awareness of LSDs, support accurate diagnosis, assist with treatment access, knowledge of treatment administration, and disease management	Create a sustainable ecosystem for individual care within a given country Create centers of excellence and referral hospitals to facilitate proper diagnosis and initiation of treatment Provide local expertise and enhance relationships that help support individuals and navigate the challenges that may be involved in reaching them
Logistical	Order processing and shipment logistical delays Extensive delays incurred due to the need for advanced provision of extensive documentation and bureaucratic challenges, for example, obtaining import permits Navigating local financial bureaucracy around donations, including import taxes, duty regulations, and cost exemptions Additional lead time needed for testing, release, shipping, and packaging products in view of cold-chain considerations During the COVID-19 pandemic, many countries closed their borders, thereby disrupting the medication supply for many individuals	Partnerships with other humanitarian and patient organizations globally Coordinate with NGOs in order to obtain import permits, tax waivers, and to better understand bureaucratic obstacles Integrate Humanitarian Program activity into all business processes, including demand forecasting, supply planning, shipping, and trade compliance Find creative solutions to ensure uninterrupted treatment for individuals (eg, delivery via road rather than air, cargo reallocation of product between hospitals, higher shipment quantity to reduce number of shipments); critical during COVID-19 pandemic and times of country crisis
Regulatory	The need to work with local government, healthcare providers, and key stakeholders to establish sustainable healthcare services for individuals with LSDs that are compliant to local regulatory and/or government requirements from diagnosis through treatment administration and follow-up	Involve all stakeholders, including external (patients, physicians, government, patient association groups) and internal team members at a global, regional, and local level
Program sustainability and unmet needs	Ensuring program is sustainable Addressing unmet needs to specific populations due to program limitations (eg, lack of treatment access for newly diagnosed adults with Gaucher disease)	Active commitment and engagement from company leadership Engage with all local stakeholders and clearly communicate the program benefits and requirements Listen to the rare-disease community, recognize that there are unmet needs, and continue to evaluate program product offerings and criteria
Cultural and personal	Ensuring respect of cultural beliefs Recognizing spectrum of disease stigma	Rely on ethicists, religious leaders, and local expertise to respectfully navigate various cultural beliefs (eg, Islamic countries such as Egypt, where parents want their sons to be treated over their daughters) Partner with international patient associations when patient voice is needed and locally is not possible due to disease stigma

LSD, lysosomal storage disease; NGO, non-governmental organization

## Reflection and vision for the future

The Humanitarian Program is an exemplar of the rare-disease community coalescing with Sanofi Genzyme’s core mission to serve one person at a time, for individuals everywhere [17]. It started as a bold vision to provide treatment access, help build sustainable healthcare systems, and improve the lives of people with LSDs. Over 3 decades it has evolved and, despite facing challenges and an ever-changing landscape, has remained true to its mission. The program has increased awareness and skills in managing LSDs, shortened diagnostics journeys, and provided treatment access, giving individuals with rare diseases the opportunity to enjoy healthier lives. Importantly, the evolution of humanitarian programs and transformation of lives has generally led to the development of interest and expertise in managing individuals affected by other rare diseases. The program has also highlighted the importance of cultural practices. For example, in India, where populations with rare diseases are not a healthcare priority, the disproportionate disease burden borne by these individuals is further compounded by consanguineous marriages. This has necessitated the introduction of culturally sensitive genetic counseling, which has been embraced by these populations, and reduced the risk of disease in offspring.

## Conclusion

The Sanofi Genzyme Humanitarian Program, founded to address the unmet needs of individuals with LSDs who previously had no available treatment options, represents one of the longest-running CAPs for LSD treatment. Providing sustainable global access to therapy, it has paved the way for other manufacturers of ERTs to engage in humanitarian aid, and has helped to overcome the world’s fear of “new and unknown” orphan drugs. The program has seen the successful implementation of treatment for people with rare complex LSDs, as well as improving disease awareness and helping physicians to build local capabilities. Additionally, individuals enrolled in the program have been empowered to become advocates, building patient organizations with national and global reach.

A key success has been maintaining an open-ended vision to provide treatment for rare diseases in underserved communities, which has resulted in its evolution from a charitable program for a single LSD to one that supports the provision of free treatment for five different LSDs to communities globally.

## Data Availability

The datasets generated and analyzed during the current study are not publicly available due to patient privacy and confidentiality. Anonymized data can be made available upon reasonable request.

## Abbreviations

AIDS: Acquired immune deficiency syndrome
CAP: Charitable access program
ERT: Enzyme replacement therapy
FD: Fabry disease
GD: Gaucher disease
HOPE: Health Opportunities for People Everywhere
JAPAC: Japan and Asia–Pacific
LATAM: Latin America
LSD: Lysosomal storage disorder
ME: Middle East
MPS: Mucopolysaccharidosis
NGO: Non-government organizations
PD: Pompe disease
US: United States
